# Supplementary material for: The Promoter Regions of Intellectual Disability-Associated Genes Are Uniquely Enriched in LTR Sequences of the MER41 Primate-Specific Endogenous Retrovirus: An Evolutionary Connection Between Immunity and Cognition
Source: Front Genet. 2019 Apr 12;10:321. doi: 10.3389/fgene.2019.00321 (PMC6473030; doi:10.3389/fgene.2019.00321)
Supplement: Supplementary file 6 [file Table_6.DOCX]

**METHODS**

We used the UniProt-provided web tool “Align” (<https://www.uniprot.org/align/>) to perform protein sequences comparisons between humans and chimpanzees. This site provides an easy way to identify amino acid dissimilarities and to determine whether or not such dissimilarities are located in sequences that are known or predicted to exert specific functions. When different isoforms of chimpanzee ortholog proteins were indexed, only the isoform displaying the highest % of homology with the reference human protein was analyzed. We did not take into account amino acid substitutions considered as not translating into dissimilar biochemical properties according to the UniProt align webtool. Also, dissimilarities that are indeed natural variants reported in humans were not accounted for humans vs chimps differences. Finally, dissimilarities occurring in “coiled coil” sequences were not considered as functionally-relevant except when a specific function was assigned to such coiled coil regions.

**RESULTS**

For each protein analyzed, are shown the % of homology between humans and chimpanzees as well as, when applicable, the number of amino acid dissimilarities and their localization in a sequence with known or predicted function. This analysis was performed on immune-related TFs that bind MER41 LTRs in the promoter regions of cognition-related (ID-associated) genes.

*IMMUNE-RELATED TRANSCRIPTION FACTORS BINDING MER41 LTRs IN THE PROMOTER REGIONS OF COGNITION-RELATED (ID-ASSOCIATED) GENES*

**Human YY1 (UniProt ID: P25490): 99.7% homology with Pan troglodytes closest ortholog (UniProt ID: K7DAI9)**

No functionally-relevant dissimilarity

**Human STAT3 (UniProt ID: P40763) 100% homology with Pan troglodytes closest ortholog (UniProt ID: A0A2I3SFL1)**

**Human NFKB1 (UniProt ID: P19838): 100% homology with Pan troglodytes closest ortholog (UniProt ID: A0A2J8MAX5)**

**Human STAT1 (UniProt ID: P42224): 100% homology with Pan troglodytes closest ortholog (UniProt ID: K7BS59)**

**Human CEBPB (UniProt ID: P17676): 100% homology with Pan troglodytes closest ortholog (UniProt ID: K7CMR0)**
